# Publisher Correction: Representational dynamics during extinction of fear memories in the human brain

**DOI:** 10.1038/s41562-025-02305-3

**Published:** 2025-09-04

**Authors:** Daniel Pacheco-Estefan, Antoine Bouyeure, George Jacob, Marie-Christin Fellner, Katia Lehongre, Virginie Lambrecq, Valerio Frazzini, Vincent Navarro, Onur Güntürkün, Lu Shen, Jing Yang, Biao Han, Qi Chen, Nikolai Axmacher

**Affiliations:** 1 Department of Basic, Developmental and Educational Psychology, Faculty of Psychology, Autonomous University of Barcelona, Barcelona, Spain; 2 Department of Neuropsychology, Institute of Cognitive Neuroscience, Faculty of Psychology, Ruhr University Bochum, Bochum, Germany; 3 Sorbonne Université, Paris Brain Institute – Institut du Cerveau, ICM, INSERM, CNRS, APHP, Pitié-Salpêtrière Hospital, Paris, France; 4 AP-HP, Département de Neurophysiologie, Hôpital Pitié-Salpêtrière, DMU Neurosciences, Paris, France; 5 AP-HP, Epilepsy Unit, Pitié-Salpêtrière Hospital, DMU Neurosciences, Paris, France; 6 AP-HP, Center of Reference for Rare Epilepsies, Pitié-Salpêtrière Hospital, Paris, France; 7 Department of Biopsychology, Institute of Cognitive Neuroscience, Faculty of Psychology, Ruhr University Bochum, Bochum, Germany; 8 Center for Studies of Psychological Application, and Guangdong Key Laboratory of Mental Health and Cognitive Science, South China Normal University, Guangzhou, China; 9 School of Psychology, South China Normal University, Guangzhou, China; 10 Key Laboratory of Brain, Cognition and Education Sciences, Ministry of Education, Beijing, China

**Keywords:** Extinction, Fear conditioning

Correction to: *Nature Human Behaviour* 10.1038/s41562-025-02268-5, published online 5 August 2025.

In the version of this article initially published, due to a processing error, instances of stimulus condition “CS–” appeared in error as “CS– –” throughout the text and figure captions. The errors have been corrected in the HTML and PDF versions of the article.

