# Supplementary material for: Representational dynamics during extinction of fear memories in the human brain
Source: Nat Hum Behav. 2025 Aug 5;10(1):29–48. doi: 10.1038/s41562-025-02268-5 (PMC12846920; doi:10.1038/s41562-025-02268-5)
Supplement: Supplementary file 1 — Supplementary Notes 1–6 and References. [file 41562_2025_2268_MOESM1_ESM.pdf]

nature human behaviour

Supplementary information https://doi.org/10.1038/s41562-025-02268-5

Representational dynamics during extinction of fear memories in the human brain

In the format provided by the authors and unedited

Table of content

Supplementary Notes 1-6

Supplementary References

Supplementary Note 1. No differences in coordination of item stability and context specificity across cue types.

We computed item stability and context specificity separately for each of our cue types (CS++, CS+- and CS--), focusing on the ROIs where we observed significant effects in the analysis including all trials during extinction. We then assessed whether coordination differed between CS+ vs CS- trials, and between CS++ vs CS+- trials. We performed the analysis across all time points in the temporal generalization map and assessed statistical significance via cluster-based permutation statistics, shuffling the condition labels 1,000 times. We only considered significant those time-by-time clusters whose summed t-values ranked above the 95th percentile of the null distribution.

In most analyses, we did not observe large clusters in the temporal generalization maps, as observed in the resulting p-values presented in the table below (corresponding to the rank of these clusters within the distribution of surrogate cluster). Thus, while our main results indicate that item stability differed between experimental conditions in the TMP and the AMY (Figure 3), the coordination of item stability across brain regions did not differ between conditions.

Analysis	Trial / Region	CS+ vs CS-	CS++ vs CS+-
Item stability	TMP-AMY	N= 22, p = 0.69	N = 19, p = 0.69
	TMP-HPC	N= 23, p = 0.8	N = 21, p = 0.38
	TMP-lPFC	N= 17, p = 0.51	N = 16, p = 0.81
	TPM-OFC	N= 9, p = 0.59	N = 9, p = 0.93
	AMY-HPC	N= 24, p = 0.34	N = 23, p = 0.86
Context specificity	lPFC-AMY	N = 14, p = 0.91	N = 14, p = 0.95
	lPFC-TMP	N= 17, p = 0.21	N = 16, p = 0.64

Summary table presents the results of the contrasts between CS+ and CS- trials, as well as between CS++ and CS+- trials, in the analyses of the coordination of both item stability and context specificity between regions. Analyses were conducted at all time points in the temporal generalization maps, and the reported p-values reflect the rank of the largest observed cluster in relation to a null distribution constructed by shuffling the condition labels. The number of participants (N) included in each analysis is also indicated.

Supplementary Note 2. No differences in correlations of item stability and context specificity with AMY_{THETA} across cue types.

To assess differences in the correlations between AMY_{THETA} and item stability or context specificity across conditions, we extracted a single-trial metric of AMY_{THETA} based on the cluster of significant differences between CS+ and CS- items observed during extinction (Figure 2; see as schematic depiction in Figure 5A, left). We separately z-scored this metric for CS+ and CS- trials, in order to avoid any spurious correlation of single-trial values driven by main condition differences. In addition, we averaged item stability and context specificity across time in the time period of AMY_{THETA} effects in each trial and each ROI. We correlated AMY theta power with item stability and context specificity across trials, separately in our three trial types (CS++, CS+- and CS--), and assessed condition differences using a one-way repeated measures ANOVA.

In the item stability analysis, our results did not reveal any significant difference between conditions in the AMY ($F(2, 44) = 1.15$, $p = 0.32$, $\eta p^2 = 0.05$). Post-hoc tests revealed that none of the pairwise comparisons showed differences, even at an uncorrected level (all $p > 0.05$). Similarly, in the HPC, correlations did not differ between conditions ($F(2, 34) = 0.2$, $p = 0.82$, $\eta p^2 = 0.01$) and pairwise comparisons were not significantly different (all $p > 0.05$). Finally, the analysis of lPFC context specificity revealed no significant differences between conditions ($F(2, 22) = 0.91$, $p = 0.42$, $\eta p^2 = 0.07$), and no significant differences in the pairwise post hoc tests (all $p > 0.05$).

These results demonstrate that the observed trial-level correlations between AMY_{THETA} and item stability in AMY and HPC (Figure 5) did not differ across trial types. In addition, they show that the correlations between AMY_{THETA} and $lPFC_{CONTEXT}$ are not significantly different across conditions. Together, these findings suggest that AMY theta oscillations play a role in the representation of cues and contexts throughout the extinction network irrespective of their valence.

Supplementary Note 3. Reinstatement analyses across cue types

We compared the reinstatement values between our three trial types (CS++, CS+-, CS--), separately for acquisition-to-test and extinction-to-test reinstatement, in the brain regions where we observed significantly higher item-stability for the CS+ as compared to the CS- trials during extinction, i.e., the AMY and the TMP. In addition, we compared the differential reinstatement of memory traces (acquisition-to-test minus extinction-to-test) across these conditions. In a separate analysis, we investigated whether the amount of acquisition-to-test reinstatement differed from the amount of extinction-to-test reinstatement for any of the categories.

For the first analysis, we assessed possible condition effects on reinstatement during time windows of 500ms during acquisition/extinction and test, sliding in 50ms (90% overlap). Only matching time-windows were considered for this analysis because a temporal generalization analysis would not be possible for the differential reinstatement, which involves time periods during three phases (acquisition, extinction, and test). We averaged across trials and across time within each 500ms window for each participant and performed one-way repeated-measures ANOVAs at each time bin to assess statistical significance. In the AMY, we did not observe any significant differences between our three conditions in the acquisition-to-test reinstatement (all time bins: $F < 1.73$, $p > 0.05$), the extinction-to-test reinstatement (all time bins: $F < 2.8$, $p > 0.05$) or in the differential reinstatement analysis (all time bins: $F < 2.54$, $p > 0.05$), even before applying correction for multiple comparisons. Similarly, in the TMP, we found no significant condition differences, even at an uncorrected level, for acquisition-to-test reinstatement (all time bins: $F < 1.4$, $p > 0.05$), extinction-to-test reinstatement (all time bins: $F < 2.53$, $p > 0.05$) or in the differential reinstatement analysis (all time bins: $F < 2.42$, $p > 0.05$; see Supplementary Figure 1). These results suggest that reinstatement does not differ between conditions.

In the second analysis, we compared the magnitude of acquisition-to-test and extinction-to-test reinstatement separately for the CS++, the CS+- and the CS-- trials. We focused on the time periods where significant item-stability effects were observed in each region (AMY: 1.25-1.5s; TMP: 0.65-1s). None of the cue types showed significant differences between acquisition-to-test and extinction-to-test reinstatement in the TMP (CS++: $t(25)= -1.11$; $p = 0.28$; CS+-: $t(25)= 0.647$; $p = 0.52$; CS--: $t(25)= 1.59$; $p = 0.12$), or in the AMY (CS++: $t(30)= -0.092$; $p = 0.93$; CS--: ($t(30)= -0.352$; $p = 0.728$; CS+-: $t(30)= 1.72$; $p = 0.096$).

Supplementary Note 4: Paradigm main features and rationale

The experimental paradigm we conducted is novel and its unique combination of features has not been validated in previous studies. However, each of the design decisions we made was based on practical and theoretical considerations derived from the fear conditioning literature. We below present a rationale for each of these design decisions.

First, we employed a multi-cue conditioning protocol in which one stimulus (CS+) predicts the unconditioned stimulus (US), while another (CS−) does not. This approach is the most commonly employed in human fear conditioning studies and differs from paradigms in which a single cue is first associated with a US and later extinguished[1].

Second, like previous research investigating the representational dynamics of fear learning [2], we used natural images as conditioned stimuli rather than auditory stimuli—more employed in animal research—or simplified shapes[3–5]. Our approach is not only arguably more ecologically valid but also better suited to capture cue representations across several sensory areas including association cortices.

Third, our study follows an ABC design, in which acquisition occurs in one context, extinction in another, and testing in a novel third context. This structure is commonly employed in both rodent and human studies that examine the role of context in fear extinction[6–11]. However, it differs from other human studies that have used ABA[12] or ABB[6] paradigms to investigate context-dependent fear extinction. We employed an ABC paradigm because we aimed to investigate whether the re-occurrence of fear memories during the final test phase was determined by the similarities of internal (i.e., neural) representations of contexts during this phase to context representations during either the acquisition or the extinction phase. This differs from ABA and ABB paradigms in which the presented contexts during the test phase are either identical to the contexts during acquisition (ABA) or during extinction (ABB).

Notably, while our paradigm follows an ABC structure, our design includes the presentation of several distinct yet thematically related context videos in each experimental phase (e.g., videos of sea landscapes). This was done to facilitate the analysis of context-specific representations using Representational Similarity Analysis (RSA), which requires the presentation of multiple instances of a particular context. In particular, as stated in our hypothesis, we aimed to evaluate the generalization of context representations during acquisition and their distinctiveness during extinction.

Fourth, we applied a partial reinforcement rate of 50%[2,13–15]. While some studies have used slightly higher rates, such as 60%[16,17], 62.5% [10], or 66%[18], we opted for 50% to promote a more gradual acquisition and extinction of fear associations (note that both processes are affected by this choice). Slower rates also reduce the risk of habituation to

the unconditioned stimulus (US), which was particularly important in our study given the relatively mild US we employed (see next point).

Fifth, in our paradigm, the US consisted of a human female face with a neutral expression following CS− items and a fearful expression following (50% of) CS+ items. Presentation of the fearful face following CS+ items was, in addition, paired with a loud scream. The choice of using emotional facial expressions was informed by studies suggesting that negative facial expressions increase resistance to extinction compared to neutral faces[19–21]. Similarly, pairing this face with a scream was grounded on previous fear conditioning research in sensitive populations such as infants and patients[22,23]. Moreover, the face-scream protocol has been shown to produce similar—though milder—responses in fear-potentiated startle and self-reported fear ratings in healthy participants as compared to electric shocks[24].

Sixth, to strengthen the association between the CS and US, we incorporated a narrative element, or "cognitive embedding". Previous research has shown that semantically or perceptually meaningful CS-US pairings are learned more effectively and are more resistant to extinction than arbitrary associations[23,25]. In our paradigm, we introduced the character of Nina, a backpacker who travels the world and stays in hotels where electric devices occasionally malfunction. Pictures of these electric devices were used as our CS (a fan, a hairdryer and a toaster). The negative emotional expression of “Nina’s” face, paired with the scream, aligns with this narrative.

Seventh, we used videos to represent contextual information rather than simpler stimuli such as colored backgrounds[26], images of scenes or rooms[16,27], or more complex VR environments[28,29]. This was made to enhance realism and reinforce the narrative of a traveler visiting diverse regions with distinct landscapes and climates while avoiding the difficulties of conducting VR research in clinical populations. In addition, we used videos since previous research has shown that their neural representations can be particularly well tracked in human electrophysiological data[30].

Eighth, as in various human fear conditioning studies that rely on self-reports rather than physiological measures, we assessed participants' expectancy ratings of safety and threat rather than collecting electrodermal activity data[31,32]. This was guided by both ethical and practical considerations, particularly given the constraints of conducting research with implanted epilepsy patients, and to emphasize the role of conscious awareness in learning and extinction.

Ninth, our testing phase occurred shortly after extinction without introducing additional delays. This decision was driven by the limited availability of patients in the clinical context. We acknowledge that some studies have implemented delays between extinction and testing to evaluate the stability of extinction in the long-term[11,27], but also highlight that several others have applied immediate testing (e.g. refs. [2,33]).

Finally, due to the time constraints of conducting research with implanted patients, we did not conduct individualized aversiveness testing for the US. Instead, as in previous studies [5,34], we selected a notoriously aversive stimulus—a loud scream—and adjusted its duration and intensity to a highly unpleasant level (1s, ~85dB).

The unique and novel combination of features in our paradigm—including the use of multiple, thematically related videos as contextual stimuli, the ABC structure, the 50% reinforcement rate, and the embedding of the experiment into a plausible narrative—has not been validated in previous research. However, as we have discussed, these methodological choices were carefully informed by the fear conditioning literature in humans. Notably, a very similar version of this paradigm, developed by our group for an fMRI study, includes the same stimuli, a very similar overall structure, and the same cover story but was conducted in healthy participants and employed a different US (electric shocks)[35]. While these data are beyond the current study, they provide an additional validation of our core paradigm choices.

Supplementary Note 5: Different electrode configurations did not affect volume of neural tissue sampled and main results

In our study, data were collected using three types of electrodes: two different types in Paris (micro-macro: 21 electrodes; macro-only: 176 electrodes) and one from Guangzhou (434 electrodes). These electrodes have variable surface area and inter-contact distances (Paris micro-macro: 1.6/3-7mm; Paris macro-only: 1.7/5mm; Guangzhou: 2/3.5mm). Additionally, within a single micro-macro electrode in Paris, the first and second contacts are separated by 3 mm, while subsequent contacts (e.g., 2 to 3, 3 to 4) are spaced 7 mm apart.

Differences in electrode configurations, combined with our use of a bipolar referencing scheme, might have affected the total volume of neural tissue sampled in our two patient groups in Paris and Guangzhou. However, it is unlikely that such differences affected our main results for several reasons. First, we only included electrodes located in gray matter, which removed a large number of contact points within each electrode (~50%). The removal of many electrode contacts introduced variability in sampling density, affecting both patient cohorts similarly. Second, a higher sampling density does not necessarily equate to more information, as closely spaced electrodes in iEEG often capture activity from the same sources and exhibit similar neurophysiological profiles. We note that most of our analyses involved averaging electrode activity in each subject independently before performing statistical comparisons at the group level, which further reduces the risk that sampling density differences systematically biased our findings. Third, many of our key findings were observed in the AMY, which had the lowest sampling density overall, with a mean number of electrodes per subject of 2.56 ± 1.39. Notably, 10 out of 22 patients had only a single electrode in the AMY, making it unlikely that inter-electrode distances or contact surface area significantly influenced our results in this region. Fourth, the inter-contact spacing of the first two electrodes in the micro-macro arrays from Paris (3 mm) closely resembles that of the electrode configurations used in Guangzhou (3.5 mm), minimizing potential sampling density differences in these electrodes.

We performed two control analyses to corroborate that different electrode configurations in our patient's cohort did not affect the density of neural tissue sampled. First, we computed the Delaunay triangulation of electrode configurations, a method that quantifies electrode density by forming triangles between contact points and calculating the mean triangle area. Focusing on our largest and more densely implanted ROI (the TMP) we evaluated triangulation in the left and right hemispheres in each subject independently. Only subjects with at least three electrodes in the TMP (N=36) were included in this analysis, as a minimum of three electrodes is required to form triangles. Statistical significance was assessed using a two-way ANOVA with "Recording site" (Guangzhou/Paris) and "Hemisphere" as factors. Results showed no significant difference in mean triangle area between hemispheres ($F(1,32) = 2.61$, $p = 0.11$, $\eta p^2 =$

0.07) or between patient cohorts ($F(1,32) = 0.96$, $p = 0.33$, $\eta p^2 = 0.03$). These findings indicate that although electrode spacing varied due to differences in electrode configurations, the overall sampling density remained consistent across our two patient groups.

To further corroborate that the different electrode configurations did not affect our results, we conducted our oscillatory power analyses in the AMY separately for Guangzhou and Paris patients. We specifically focused on the significant cluster observed in the main analysis contrasting "current" valence (CS+ vs CS-) during extinction. We observed the same pattern of results across our two cohorts of patients, with higher theta power for CS- as compared to CS+ items during extinction (Guangzhou: $t(19)= -4.32$, $p = 0.0004$, Cohen's $d = -1.24$, CI: [-2.08, -0.58]; Paris: $t(11)= 2.93$, $p = 0.014$, Cohen's $d = -1.28$, CI: [-2.64, -0.27]). Taken together, the results of these analyses suggest that our two patient cohorts did not show prominent differences in the neural tissue sampled in the TMP, and that the AMY theta power results during extinction were consistent in our two patient groups (see Supplementary Figure 2).

Finally, we highlight that recent years have seen an increase of multi-center iEEG research, which involves collecting and analyzing data from multiple epilepsy centers to generate larger and more diverse datasets. Several studies have successfully integrated multi-center iEEG data despite variations in electrode configurations, including even combination of subdural strips and grids with depth electrodes, which differ in regard to sampling density even more than different types of depth electrodes do[36–38]. While there is currently no established consensus or guideline on how to address sampling differences in the literature, we believe this is an important topic of research in the future, as multi-center iEEG studies are becoming more prominent.

Supplementary Note 6: Connectivity analysis using the weighted phase lag index

Conceptually, we were predominantly interested in investigating the representations of contexts and items during fear learning and extinction. For this reason, we employed metrics of representational coordination rather than standard connectivity analyses in order to investigate the propagation of representational signatures throughout the fear and extinction network. However, we acknowledge that connectivity metrics could offer complementary insights into the neural mechanisms of fear extinction.

Over the past decades, several connectivity metrics have been developed and applied. One of the first to be proposed was the Phase Locking Value (PLV)[39], which quantifies the consistency of phase angle differences between two signals. The PLV has been widely applied in cognitive neuroscience[40–42], but this metric is highly susceptible to volume conduction since it includes apparent connectivity at phase differences at zero lag. Volume conduction refers to the phenomenon where electrical activity from neuronal sources propagates almost instantaneously through brain tissues, leading to signal spread. This effect can artificially inflate metrics of connectivity in iEEG, as different electrodes might capture activity from the same neurophysiological source.

To circumvent the problem of volume conduction, several metrics based on phase lag were developed in the last years. In particular, phase-based connectivity measures were established that ignore zero phase-lag connectivity, including the phase-slope index[43], the phase lag index[44], and the weighted phase-lag index[45]. The Phase Lag Index (PLI) quantifies asymmetries in phase lead/lag, ignoring synchronized activity at zero or 180° phase lag—which is often the result of volume conduction rather than true connectivity. The weighted PLI (wPLI) assigns greater weight to larger phase differences by considering the magnitude of the imaginary component of the cross-spectrum, effectively suppressing small phase differences that may result from volume conduction. By improving robustness against noise as compared to the PLI and minimizing false connectivity, wPLI has become a widely employed metric for mapping functional networks in EEG/iEEG research[45,46].

To complement our representational coordination analyses and provide a more comprehensive description of how fear extinction signals propagate throughout the fear and extinction network, we assessed the wPLI between pairs of ROIs. We specifically focused on the ROIs where we observed significant coordination in our item stability (Figure 3D) or context specificity (Figure 4D) analyses. These included AMY-TMP, AMY-HPC, AMY-lPFC, TMP-HPC, TMP-OFC, and TMP-lPFC. We computed wPLI across trials for each pair of electrodes in their correspondent ROI pairs, focusing on the theta (4–8 Hz) frequency band, as this was the frequency range where we observed the most prominent effects in the AMY in our data. Notably, phase-based connectivity in the theta range has been associated with long-range neural communication[47]. We specifically

selected the time periods where we observed significant coordination effects in the original analysis (see table below).

We computed wPLI for all electrode pairs within predefined ROI pairs, separately for each subject and each of our three cue types (CS++, CS+-, and CS--). In subjects with bi-hemispheric implants, we computed wPLI for channel pairs within the same hemisphere only. wPLI metrics obtained for each electrode pair were averaged across electrodes in each subject before performing statistical comparisons. Significant differences were assessed with a repeated measures one-way ANOVA.

Our results did not reveal any significant differences in wPLI during the time periods of significant coordination in any of the ROI pairs even at an uncorrected level (see table below).

**ROIs**	**Time period**	**ANOVA results**
AMY-TMP	0-1.75s	$F(2,24) = 2.22$, $p = 0.12$, $N = 27$
AMY-HPC	0.5-1.75s	$F(2,20) = 1.08$, $p = 0.34$, $N = 23$
AMY-lPFC	1-1.65s	$F(2,10) = 0.16$, $p = 0.85$, $N = 13$
TMP-HPC	0-1.45	$F(2,22) = 0.09$, $p = 0.91$, $N = 25$
TMP-OFC	0.15-1s	$F(2,7) = 0.72$, $p = 0.5$, $N = 10$
TMP-lPFC	0-0.8s	$F(2,14) = 0.24$, $p = 0.78$, $N = 17$

The absence of significant differences in our phase-based connectivity analysis suggests that the observed coordination in representational signals in our study is not linked to phase-based connectivity in the theta frequency range. However, we acknowledge that the space of possible connectivity analyses is vast, and our metric of representational coordination may align more closely with other forms of connectivity, such as power-based or causal connectivity, which could be investigated more comprehensively in the future.

Supplementary References

1. Lonsdorf, T. B. *et al.* Don't fear 'fear conditioning': Methodological considerations for the design and analysis of studies on human fear acquisition, extinction, and return of fear. *Neuroscience & Biobehavioral Reviews* **77**, 247–285 (2017).
2. Visser, R. M., Scholte, H. S., Beemsterboer, T. & Kindt, M. Neural pattern similarity predicts long-term fear memory. *Nature Neuroscience* **16**, 388–390 (2013).
3. Chen, S. *et al.* Theta oscillations synchronize human medial prefrontal cortex and amygdala during fear learning. *Science Advances* **7**, eabf4198 (2021).
4. Neumann, D. L., Lipp, O. V. & Cory, S. E. Conducting extinction in multiple contexts does not necessarily attenuate the renewal of shock expectancy in a fear-conditioning procedure with humans. *Behaviour Research and Therapy* **45**, 385–394 (2007).
5. Schmitz, A. & Grillon, C. Assessing fear and anxiety in humans using the threat of predictable and unpredictable aversive events (the NPU-threat test). *Nature protocols* **7**, 527–532 (2012).
6. Balooch, S. B., Neumann, D. L. & Boschen, M. J. Extinction treatment in multiple contexts attenuates ABC renewal in humans. *Behaviour Research and Therapy* **50**, 604–609 (2012).
7. Corcoran, K. A. & Maren, S. Hippocampal Inactivation Disrupts Contextual Retrieval of Fear Memory after Extinction. *J. Neurosci.* **21**, 1720 (2001).
8. Corcoran, K. A. & Maren, S. Factors regulating the effects of hippocampal inactivation on renewal of conditional fear after extinction. *Learning & Memory* **11**, 598–603 (2004).

9. Effting, M. & Kindt, M. Contextual control of human fear associations in a renewal paradigm. *Behaviour Research and Therapy* **45**, 2002–2018 (2007).
10. Hermann, A., Stark, R., Milad, M. & Merz, C. Renewal of conditioned fear in a novel context is associated with hippocampal activation and connectivity. *Social cognitive and affective neuroscience* **11**, 1411–1421 (2016).
11. Milad, M. R., Orr, S. P., Pitman, R. K. & Rauch, S. L. Context modulation of memory for fear extinction in humans. *Psychophysiology* **42**, 456–464 (2005).
12. Krisch, K. A., Bandarian-Balooch, S. & Neumann, D. L. Effects of extended extinction and multiple extinction contexts on ABA renewal. *Learning and Motivation* **63**, 1–10 (2018).
13. Dunsmoor, J. E., Bandettini, P. A. & Knight, D. C. Impact of continuous versus intermittent CS-UCS pairing on human brain activation during Pavlovian fear conditioning. *Behavioral neuroscience* **121**, 635 (2007).
14. Dunsmoor, J. E., Martin, A. & LaBar, K. S. Role of conceptual knowledge in learning and retention of conditioned fear. *Biological Psychology* **89**, 300–305 (2012).
15. Dunsmoor, J. E., Ahs, F., Zielinski, D. J. & LaBar, K. S. Extinction in multiple virtual reality contexts diminishes fear reinstatement in humans. *Neurobiology of Learning and Memory* **113**, 157–164 (2014).
16. Battaglia, S., Garofalo, S. & di Pellegrino, G. Context-dependent extinction of threat memories: influences of healthy aging. *Scientific Reports* **8**, 12592 (2018).
17. Milad, M. R. *et al.* Recall of Fear Extinction in Humans Activates the Ventromedial Prefrontal Cortex and Hippocampus in Concert. *Biological Psychiatry* **62**, 446–454 (2007).

18. Dunsmoor, J. E. & Murphy, G. L. Stimulus Typicality Determines How Broadly Fear Is Generalized. *Psychol Sci* **25**, 1816–1821 (2014).
19. Mineka, S. & Öhman, A. Phobias and preparedness: the selective, automatic, and encapsulated nature of fear. *Biological Psychiatry* **52**, 927–937 (2002).
20. Ney, L. J., O'Donohue, M. P., Lowe, B. G. & Lipp, O. V. Angry and fearful compared to happy or neutral faces as conditional stimuli in human fear conditioning: A systematic review and meta-analysis. *Neuroscience & Biobehavioral Reviews* **139**, 104756 (2022).
21. Öhman, A. & Öst, L.-G. Animal and social phobias: Biological constraints on learned fear responses. *Theoretical issues in behavior therapy* 123–175 (1985).
22. Glenn, C. R. *et al.* The development of fear learning and generalization in 8–13 year-olds. *Developmental Psychobiology* **54**, 675–684 (2012).
23. Hamm, A. O., Vaitl, D. & Lang, P. J. Fear conditioning, meaning, and belongingness: A selective association analysis. *Journal of Abnormal Psychology* **98**, 395–406 (1989).
24. Glenn, C. R., Lieberman, L. & Hajcak, G. Comparing electric shock and a fearful screaming face as unconditioned stimuli for fear learning. *International Journal of Psychophysiology* **86**, 214–219 (2012).
25. Garcia, J. & Koelling, R. A. Relation of cue to consequence in avoidance learning. *Psychonomic Science* **4**, 123–124 (1966).
26. Kalisch, R. *et al.* Context-Dependent Human Extinction Memory Is Mediated by a Ventromedial Prefrontal and Hippocampal Network. *J. Neurosci.* **26**, 9503 (2006).

27. Xia, Y. *et al.* Measuring human context fear conditioning and retention after consolidation. *Learning & Memory* **30**, 139–150 (2023).

28. Baas, J. M., Nugent, M., Lissek, S., Pine, D. S. & Grillon, C. Fear conditioning in virtual reality contexts: a new tool for the study of anxiety. *Biological Psychiatry* **55**, 1056–1060 (2004).

29. Glotzbach-Schoon, E. *et al.* Enhanced discrimination between threatening and safe contexts in high-anxious individuals. *Biological Psychology* **93**, 159–166 (2013).

30. Michelmann, S., Bowman, H. & Hanslmayr, S. The Temporal Signature of Memories: Identification of a General Mechanism for Dynamic Memory Replay in Humans. *PLOS Biology* **14**, e1002528 (2016).

31. Bandarian Balooch, S. & Neumann, D. L. Effects of multiple contexts and context similarity on the renewal of extinguished conditioned behaviour in an ABA design with humans. *Learning and Motivation* **42**, 53–63 (2011).

32. Boddez, Y. *et al.* Rating data are underrated: Validity of US expectancy in human fear conditioning. *Journal of Behavior Therapy and Experimental Psychiatry* **44**, 201–206 (2013).

33. Vansteenwegen, D. *et al.* The repeated confrontation with videotapes of spiders in multiple contexts attenuates renewal of fear in spider-anxious students. *Behaviour Research and Therapy* **45**, 1169–1179 (2007).

34. Wehrli, J. M., Xia, Y., Gerster, S. & Bach, D. R. Measuring human trace fear conditioning. *Psychophysiology* **59**, e14119 (2022).

35. Bouyeure, A. *et al.* Distinct representational properties of cues and contexts shape fear learning and extinction. (2025) doi:10.7554/elife.105126.1.

36. Bernabei, J. M. *et al.* Quantitative approaches to guide epilepsy surgery from intracranial EEG. *Brain* **146**, 2248–2258 (2023).

37. Dimakopoulos, V. *et al.* Protocol for multicentre comparison of interictal high-frequency oscillations as a predictor of seizure freedom. *Brain Communications* **4**, fcac151 (2022).

38. Henin, S. *et al.* Spatiotemporal dynamics between interictal epileptiform discharges and ripples during associative memory processing. *Brain* **144**, 1590–1602 (2021).

39. Lachaux, J.-P., Rodriguez, E., Martinerie, J. & Varela, F. J. Measuring phase synchrony in brain signals. *Human Brain Mapping* **8**, 194–208 (1999).

40. Mormann, F., Lehnertz, K., David, P. & E. Elger, C. Mean phase coherence as a measure for phase synchronization and its application to the EEG of epilepsy patients. *Physica D: Nonlinear Phenomena* **144**, 358–369 (2000).

41. Pacheco Estefan, D. *et al.* Coordinated representational reinstatement in the human hippocampus and lateral temporal cortex during episodic memory retrieval. *Nature communications* **10**, 1–13 (2019).

42. Varela, F., Lachaux, J.-P., Rodriguez, E. & Martinerie, J. The brainweb: phase synchronization and large-scale integration. *Nature reviews neuroscience* **2**, 229–239 (2001).

43. Nolte, G. *et al.* Robustly estimating the flow direction of information in complex physical systems. *Physical review letters* **100**, 234101 (2008).

44. Stam, C. J., Nolte, G. & Daffertshofer, A. Phase lag index: assessment of functional connectivity from multi channel EEG and MEG with diminished bias from common sources. *Human brain mapping* **28**, 1178–1193 (2007).

45. Vinck, M., Oostenveld, R., van Wingerden, M., Battaglia, F. & Pennartz, C. M. A. An improved index of phase-synchronization for electrophysiological data in the presence of volume-conduction, noise and sample-size bias. *NeuroImage* **55**, 1548–1565 (2011).

46. Imperatori, L. S. *et al.* EEG functional connectivity metrics wPLI and wSMI account for distinct types of brain functional interactions. *Scientific Reports* **9**, 8894 (2019).

47. Fell, J. & Axmacher, N. The role of phase synchronization in memory processes. *Nat Rev Neurosci* **12**, 105–118 (2011).
