# Supplementary material for: Representational dynamics during extinction of fear memories in the human brain
Source: Nat Hum Behav. 2025 Aug 5;10(1):29–48. doi: 10.1038/s41562-025-02268-5 (PMC12846920; doi:10.1038/s41562-025-02268-5)
Supplement: Supplementary file 2 — Reporting Summary [file 41562_2025_2268_MOESM2_ESM.pdf]

nature portfolio

Corresponding author(s): Daniel Pacheco, Nikolai Axmacher, Qi Chen

Last updated by author(s): May 8, 2025

Reporting Summary

Nature Portfolio wishes to improve the reproducibility of the work that we publish. This form provides structure for consistency and transparency in reporting. For further information on Nature Portfolio policies, see our Editorial Policies and the Editorial Policy Checklist.

Statistics

For all statistical analyses, confirm that the following items are present in the figure legend, table legend, main text, or Methods section.

n/a	Confirmed	
☐	☒	The exact sample size (*n*) for each experimental group/condition, given as a discrete number and unit of measurement
☐	☒	A statement on whether measurements were taken from distinct samples or whether the same sample was measured repeatedly
☐	☒	The statistical test(s) used AND whether they are one- or two-sided<br>*Only common tests should be described solely by name; describe more complex techniques in the Methods section.*
☐	☒	A description of all covariates tested
☐	☒	A description of any assumptions or corrections, such as tests of normality and adjustment for multiple comparisons
☐	☒	A full description of the statistical parameters including central tendency (e.g. means) or other basic estimates (e.g. regression coefficient) AND variation (e.g. standard deviation) or associated estimates of uncertainty (e.g. confidence intervals)
☐	☒	For null hypothesis testing, the test statistic (e.g. *F*, *t*, *r*) with confidence intervals, effect sizes, degrees of freedom and *P* value noted<br>*Give P values as exact values whenever suitable.*
☒	☐	For Bayesian analysis, information on the choice of priors and Markov chain Monte Carlo settings
☒	☐	For hierarchical and complex designs, identification of the appropriate level for tests and full reporting of outcomes
☒	☐	Estimates of effect sizes (e.g. Cohen's *d*, Pearson's *r*), indicating how they were calculated

Our web collection on statistics for biologists contains articles on many of the points above.

Software and code

Policy information about availability of computer code

Data collection	The experiment was programmed in Presentation (Neurobehavioral Systems, California, USA), and was deployed on Laptop computers running Microsoft Windows.
Data analysis	Behavioral and intracranial EEG data was analyzed using custom-written Matlab (R2024B) code, deposited in GitHub (https://github.com/dpachec/extinction). Several Matlab toolboxes were employed, including EEGLAB (v2021.0; https://sccn.ucsd.edu/eeglab/) and Fieldtrip (08-11-2024; https://www.fieldtriptoolbox.org/). Software employed for electrode localization included FreeSurfer (v7.4.1; https://surfer.nmr.mgh.harvard.edu/), 3D Slicer (v5.6.2; https://www.slicer.org/), SPM (v12; https://www.fil.ion.ucl.ac.uk/spm/) and BrainVisa (v5.1; https://brainvisa.info/web/).

For manuscripts utilizing custom algorithms or software that are central to the research but not yet described in published literature, software must be made available to editors and reviewers. We strongly encourage code deposition in a community repository (e.g. GitHub). See the Nature Portfolio guidelines for submitting code & software for further information.

Data

Policy information about availability of data

All manuscripts must include a data availability statement. This statement should provide the following information, where applicable:

- Accession codes, unique identifiers, or web links for publicly available datasets
- A description of any restrictions on data availability
- For clinical datasets or third party data, please ensure that the statement adheres to our policy

Anonymized intracranial EEG data supporting the findings of this study are available on the Open Science Framework at https://osf.io/xfprt/

Research involving human participants, their data, or biological material

Policy information about studies with human participants or human data. See also policy information about sex, gender (identity/presentation), and sexual orientation and race, ethnicity and racism.

Reporting on sex and gender	We collected data from both sexes in this study (self-reported), and did not evaluate differences related to sex for performing the task.
Reporting on race, ethnicity, or other socially relevant groupings	No distinctions were made regarding race or ethnicity. The experiment was conducted with different patient populations in France and China, and data from the two patient populations was aggregated in all analyses.
Population characteristics	Forty-nine patients (22 females, 28.2 ± 8.18 years) with medically intractable epilepsy participated in the study.
Recruitment	Data were collected at the Pitié Salpêtrière hospital, Paris, and the South China Normal University Hospital, Guangzhou, China. All patients were implanted for clinical purposes and participation in the study was voluntary. The study was conducted with all available patients, with no selection criteria.
Ethics oversight	The study was conducted according to the latest version of the Declaration of Helsinki and approved by the institutional review board at the Pitié Salpêtrière hospital (C11-16, C19-55 National Institute of Health and Medical Research sponsor), and by the ethics committee at the South China Normal University Hospital, Guangzhou. All patients provided written informed consent.

Note that full information on the approval of the study protocol must also be provided in the manuscript.

Field-specific reporting

Please select the one below that is the best fit for your research. If you are not sure, read the appropriate sections before making your selection.

☒ Life sciences ☐ Behavioural & social sciences ☐ Ecological, evolutionary & environmental sciences

For a reference copy of the document with all sections, see nature.com/documents/nr-reporting-summary-flat.pdf

Life sciences study design

All studies must disclose on these points even when the disclosure is negative.

Sample size	Since no prior iEEG research has specifically examined fear extinction, we based our sample size on previous studies investigating related processes, such as fear acquisition (e.g., Chen et al., Sci. Adv., 2021) and emotional memory formation (e.g., Costa et al., Nat. Commun., 2022; Zhang et al., Nat. Commun., 2024). These studies typically involved small samples (Chen et al., N = 13; Zhang et al., N = 7; Costa et al., N = 19). Our cohort of N = 49 patients substantially exceeds the sample sizes employed in these and other comparable studies.
Data exclusions	From a total of 50 subjects, one subject had to be excluded because he did not provided enough answers during the acquisition and extinction periods (12.5% and 7.8% respectively), leading to a total number of N=49 subjects.
Replication	Our study was not replicated, primarily due to the challenges of accessing iEEG patient populations. However, our relatively large sample size compared to most iEEG studies (N=49) enhances the statistical robustness of our findings.
Randomization	All participants took part in all experimental conditions in our study following a within subjects design.
Blinding	Investigators were blind to experimental conditions during data collection and during the preprocessing of the data (artifact rejection) as explained in the manuscript (Methods section).

Reporting for specific materials, systems and methods

We require information from authors about some types of materials, experimental systems and methods used in many studies. Here, indicate whether each material, system or method listed is relevant to your study. If you are not sure if a list item applies to your research, read the appropriate section before selecting a response.

Materials & experimental systems

n/a	Involved in the study
☒	☐ Antibodies
☒	☐ Eukaryotic cell lines
☒	☐ Palaeontology and archaeology
☒	☐ Animals and other organisms
☒	☐ Clinical data
☒	☐ Dual use research of concern
☒	☐ Plants

Methods

n/a	Involved in the study
☒	☐ ChIP-seq
☒	☐ Flow cytometry
☒	☐ MRI-based neuroimaging

Plants

Seed stocks	*Report on the source of all seed stocks or other plant material used. If applicable, state the seed stock centre and catalogue number. If plant specimens were collected from the field, describe the collection location, date and sampling procedures.*
Novel plant genotypes	*Describe the methods by which all novel plant genotypes were produced. This includes those generated by transgenic approaches, gene editing, chemical/radiation-based mutagenesis and hybridization. For transgenic lines, describe the transformation method, the number of independent lines analyzed and the generation upon which experiments were performed. For gene-edited lines, describe the editor used, the endogenous sequence targeted for editing, the targeting guide RNA sequence (if applicable) and how the editor was applied.*
Authentication	*Describe any authentication procedures for each seed stock used or novel genotype generated. Describe any experiments used to assess the effect of a mutation and, where applicable, how potential secondary effects (e.g. second site T-DNA insertions, mosiacism, off-target gene editing) were examined.*
